# Activity Recognition from Daily-Life Sounds Using Unsupervised Learning with Dirichlet Multinomial Mixture Models

**DOI:** 10.3390/s26051509

**Published:** 2026-02-27

**Authors:** Ken Sadohara, Natsuki Miyata

**Affiliations:** National Institute of Advanced Industrial Science and Technology (AIST), 2-3-26 Aomi, Koto-ku, Tokyo 135-0064, Japan; n.miyata@aist.go.jp

**Keywords:** activity recognition, ambient assisted living, acoustic scene classification, Dirichlet multinomial mixture model (DMM), burstiness, topic model, neural audio codec, bespoke system

## Abstract

To support ambient assisted living for the elderly living alone, we investigate a method for recognizing daily activities from household sounds. To reduce the cost of building an activity-recognition model, we adopt an unsupervised learning approach based on a Dirichlet multinomial mixture model. The model represents the generative process of neural audio codec codes conditioned on latent activities. We further extend the model to handle multiple streams of codes corresponding to different sound directions. This extension enables the formation of more accurate activity clusters, partly because code occurrence patterns exhibit burstiness. The proposed approach is expected to serve as a key component for constructing an activity recognition system that requires minimal labeled data and a small number of user inquiries.

## 1. Introduction

According to the annual report on aging society in Japan [1], the number of people aged 65 and over living alone is projected to exceed 10 million by 2040. Therefore, monitoring and supporting independent living for the elderly has become an important social issue not only for distant family members but also for local governments [2,3]. Ambient assisted living (AAL) [4] has been considered a solution for improving the quality of life of individuals, by enabling people to live healthily and independently.

In particular, activity recognition has been identified as a key technology that predicts activities of daily living (ADLs) based on signals from wearable or environmental sensors [5,6,7,8]. With the advancement of Internet of Things (IoT) technology, data acquisition from sensors has become reliable, affordable and accessible to many people. At the same time, the data analysis systems for predicting activities have become feasible because a wide range of machine learning techniques can be used to build accurate activity classifiers when sufficient data are available. However, the literature [8] has pointed out that, for activity recognition systems to be widely available, substantial challenges remain in developing systems that are deployable ‘as-is’ and semi-automatically adaptable to individuals across different environments without requiring additional resources in terms of cost, effort, or time. Because minimal interaction with experts or users is required during the adaptation process, such activity recognition systems are referred to as ‘bespoke’ systems.

For a bespoke system, a large amount of labeled data cannot be prepared because the cost of an annotation is generally high. Moreover, a model trained for a particular individual cannot be applied to other individuals particularly when only unobtrusive sensors, such as passive infrared ray (PIR) motion sensors, are available. This limitation arises because classification models depend on individual behavioral patterns and living environments. Differences in room layout lead to variations in sensor placement, and differences in household items and how they are used naturally result in variations in the daily sounds observed. Therefore, the model should be individually tailored to each user.

To keep modeling cost within reasonable bounds, unsupervised learning techniques have been identified as a promising approach [7,9]. However, in previous research, unsupervised learning has achieved only limited success in activity recognition. The authors have demonstrated that the Dirichlet multinomial mixture (DMM) models [10,11] are effective for data obtained from PIR sensors, which exhibit the burstiness property [9]. If it can be demonstrated that, under an appropriate method for discretizing acoustic signals, the distribution of discrete symbols also exhibits burstiness, then applying DMM models to household sounds becomes a promising direction. The use of the acoustic modality is helpful because, compared with PIR sensors that simply detect the location where an activity occurs, acoustic sensors can distinguish between different activities performed in the same location.

Predicting activities from ambient sound is referred to as acoustic scene analysis (ASA), which has been studied extensively. However, most existing approaches rely on supervised learning, commonly referred to as acoustic scene classification (ASC). In the context of ASC, discretization of acoustic signals, such as acoustic words [12], has been studied. To leverage recent advances in neural auto-encoders, we explore the use of a neural audio codec, which is an end-to-end neural auto-encoder that generates discretized codes as bottleneck features. In the distribution of codes obtained using the neural audio codec, we again observe a burstiness property similar to that observed in signals from PIR sensors.

**Figure 1:**
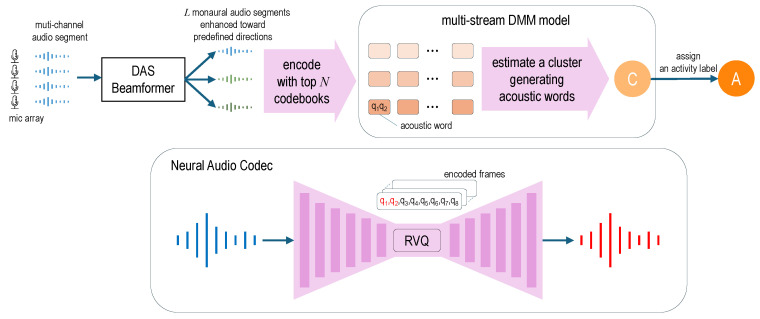
A diagram illustrating the process of assigning an activity label to a household audio segment using the proposed method. Clusters are obtained in the training phase by clustering a large amount of acoustic word sequences without activity labels. In the experiments reported in this paper, the number of streams *L* was set to three, and the number of codebooks *N* was set to two.

This observation supports the use of the DMM model for unsupervised clustering of the codes. To verify this hypothesis, we extend the DMM model to handle multiple streams of codes corresponding to different sound directions in order to utilize spatial information in multi-channel audio recordings (Figure 1). The extended model is applied to a publicly available real-life dataset, and results show that it produces more accurate clusters than other unsupervised algorithms.

The contributions of this study are as follows:

- It is shown that data encoded from daily-life sounds using a neural audio codec exhibit burstiness.
- To leverage spatial information contained in daily-life sounds for clustering, a multi-stream extension of the DMM model, as well as a Gibbs sampler  [13] suitable for estimating its parameters, is presented.
- Together with the encoder, the extended DMM model, which can capture burstiness, is shown to outperform existing unsupervised clustering methods on a real-life dataset.

By asking users which activities correspond to the obtained clusters, an activity recognition system can gradually improve its accuracy during use. This study aimed to create such a bespoke activity recognition system, and key components necessary for its realization are proposed.

The remainder of this work is organized as follows. Section 2 briefly reviews previously studied unsupervised activity recognition techniques. Section 3 describes the dataset used in this study and and presents experimental results for both supervised and unsupervised activity recognition using a neural auto-encoder, which provide an approximate upper limit and a baseline target performance for the dataset. Section 4 demonstrates the burstiness property in the dataset and presents the DMM model extended to cope with multiple streams of sensor data, together with a method for estimating model parameters. Section 5 applies the extended model to the dataset to evaluate its performance. In Section 6, we present areas for future study, and finally, in Section 7, we conclude with a summary of the results.

## 2. Related Works

Activity recognition, which identifies known activities, has been studied extensively. In contrast, the use of unsupervised learning in activity recognition, referred to as activity discovery  [14], has received comparatively less attention [5,6,7]. Research on activity discovery conducted to date has included the use of background knowledge [15] or heuristics of daily behaviors [16], the application of frequent sequence mining [14,17,18,19], and the use of probabilistic models [20,21,22,23,24]. This paper also adopts a generative probabilistic approach based on the DMM model [10,11], which has been shown to be effective for data from PIR sensors that exhibit burstiness, and to produce more accurate clusters [9] than alternative models such as latent Dirichlet allocation (LDA) models [25] and hierarchical Dirichlet process (HDP) models [26].

Activity recognition from ambient sound is commonly studied under acoustic scene classification (ASC) [27]. ASC addresses the problem of identifying the situation or environment in which a sound is generated, typically distinguishing between contexts such as ‘train’, ‘home’ or ‘meeting’. In contrast, a closely related problem, sound event detection (SED), focuses on identifying which sounds occur during specific time intervals, typically distinguishing events such as ‘birds singing’, ‘cutlery sounds’, or ‘car horn’. Because certain sound events are more likely to occur in specific acoustic scenes, ASC and SED are inherently related problems [28]. As a result, some researchers adopt multitask learning approaches to jointly address both problems [29].

On the other hand, the acoustic topic model (ATM) [12,30,31] defines a process in which acoustic words, analogues of acoustic events, are generated depending on latent acoustic topics, analogues of acoustic scenes, in a manner similar to how topic models for documents, such as the LDA models, define the generation of words conditioned on hidden document topics. Specifically, acoustic words are obtained frame by frame by clustering feature vectors extracted from audio frames, after which the acoustic topics that generate these acoustic words are estimated. Finally, an acoustic scene for an audio segment is classified based on the estimated acoustic topics of frames within the segment where the classifier is trained using supervised learning. The DMM model is also a topic model and is comparatively simpler than the LDA model. However, because of its simplicity, the acoustic topics correspond more directly to acoustic scenes and the model is able to capture burstiness.

Unsupervised ASC methods that involve clustering acoustic feature vectors have also been explored [32,33]. In [33], a neural auto-encoder was used to extract embedding vectors from the bottleneck layer. For each audio segment, a feature vector was obtained by averaging embedding vectors of all frames. Then, Spectral Clustering was applied to the set of feature vectors to obtain clusters corresponding to activities. Because the dimensionality of acoustic feature vectors is typically high, clustering in low-rank subspaces, such as Spectral Clustering, has been reported to be effective. Concatenating feature vectors obtained from different audio channels to incorporate spatial information was also shown to be effective. In the following section, these promising methods are applied to the target dataset.

With respect to low-resource ASC, data augmentation and semi-supervised learning have also been explored [34,35]. However, these approaches still require a large amount of labeled data. This paper adopts an approach that first performs unsupervised clustering and then identifies activities through a small number of questions posed to the user.

## 3. Preliminary Experiments

Prior to describing unsupervised learning with DMM models, we present results from preliminary experiments on daily activity recognition using daily-life sounds and an acoustic foundation model to narrow the experimental conditions.

### 3.1. Dataset

We use a dataset from the DCASE2018 Challenge Task5 [36], whose goal is to classify domestic activities by using multi-channel acoustic recordings. The dataset is derived from the SINS dataset [37], which contains a continuous recording of one individual living alone one week. To investigate the effectiveness of multi-channel acoustic recordings, the task provides a development dataset and an evaluation dataset, each consisting of 4-channel audio segments of 10 s recorded by microphone array nodes in a living room, together with the ground truth labels for nine activities. In this study, we use the development dataset because it allows for identification of which of the four microphone array nodes produced each audio segment. As we mentioned earlier, expensive use of microphone array nodes is considered cost-prohibitive in the target application. Therefore, this dataset is particularly suitable because it enables evaluation of daily activity recognition using a single microphone array node. Figure 1 in the study [37] illustrates the floorplan and position of the four microphone array nodes.

We do not follow the same evaluation procedure as the original competition. Instead, we first split the entire dataset into four parts, one for each microphone array node, in order to evaluate classification performance separately for each node. Each split dataset is then further divided into 64 folds to investigate the influence of labeled data availability. For these 64 folds, two types of 64-fold cross validation are conducted. The first setting, denoted as $E_{1}V_{1}T_{62}$, represents scenarios in which a relatively large amount of labeled data is available. In this setting, one fold is selected as the evaluation dataset, then the subsequent fold is used as the validation dataset, and the remaining folds are used for training. The second setting, denoted as $T_{1}V_{1}E_{62}$, represents scenarios in which only a small amount of labeled data is available. In this case, one fold is selected as the training dataset, then the next fold as the validation dataset, and the remaining folds are used for evaluation.

Another difference from the DCASE2018 Task5 is that we exclude ‘absence’ activities, which indicates that no individual is present in the living room. Distinguishing ‘absence’ activities from silence during other activities is generally difficult because of the limited availability of auditory cues. However, ‘absence’ can be detected using other sensors such as PIR sensors. Therefore, we consider only eight activities, as described in Table 1, which reports the number of labeled samples for each activity averaged across training and validation datasets under the large labeled data setting $E_{1}V_{1}T_{62}$ for Node2. The table also reports the minimum and maximum number of samples for each activity across the corresponding training and validation sets. Table 2 presents the same statistics for the small labeled data setting $T_{1}V_{1}E_{62}$ for Node2. These statistics are nearly identical across other microphone array nodes.

In the DCASE2018 challenge Task5, results for the development dataset were reported as follows: the macro-averaged F1-score of the baseline system was 84.5% and increased to 85.5% when the ‘absence’ activity was excluded [36]. The highest F1-score achieved by participating systems among participants was 90.0% [38]. As noted above, our evaluation setting differs from that of DCASE2018 Task5. Therefore, we conduct experiments under our own setting and report the corresponding results in the remainder of this section.

### 3.2. Beamforming

To utilize spatial information, audio signals, directed toward three predefined directions ($0^{\circ }$, $+30^{\circ }$ and $-30^{\circ }$), are computed using the delay-and-sum (DAS) beamforming technique. Specifically, after time delays are applied to signals of each microphone to compensate for arrival time differences from a particular direction, the delayed and aligned signals are then summed from all microphones. This process is expected to enhance direct sounds coming from the specified direction. The HARK software [39] is used to obtain audio signals enhanced toward these predefined directions. For each microphone array node and each 4-channel audio segment recorded by the node, a monaural audio signal toward one predefined direction is generated. In this manner, the entire dataset is expanded such that each original audio segment corresponds to three monaural audio recordings.

### 3.3. Neural Audio Codec

The neural audio codec [40,41] is an audio coding framework that leverages an end-to-end neural auto-encoder comprising an encoder, a bottleneck and a decoder. The auto-encoder is trained by minimizing the reconstruction loss between encoder input and decoder output. The bottleneck contains latent variables that acquire a compressed representation of the input signal. In the neural audio codec EnCodec [41] employed in this study, the bottleneck is represented by discrete codes obtained using a residual vector quantizer (RVQ) [40]. Each embedding vector produced by the final layer of the encoder is converted into a sequence of codes, which can be transmitted and subsequently decoded by a receiver using the decoder. While these codes are later used to obtain clusters of audio segments, the embedding vectors are used to train activity classifiers using supervised learning in the following section.

### 3.4. Supervised Learning

For each 10 s audio segment, 748 vectors with dimensionality 128 are obtained using EnCodec. These vectors are then averaged and normalized to produce a single feature vector of dimension 128 for each audio segment. To train classifiers on these feature vectors, we use software developed for the HEAR benchmark [42]. For each combination of hyper-parameters such as the number of hidden layers, the learning rate and the initialization method, the software trains a multi-layer perceptron using a training dataset. Among the trained models, the performing classifier is selected using a validation dataset. The selected classifier is then applied to the evaluation dataset to predict activities for all audio segments.

Classifier training and prediction are performed independently for each audio stream corresponding to the predefined directions. Final ensemble predictions across all streams are obtained in a manner similar to that described in [43]. Specifically, the output of the final softmax layers of classifiers corresponding to the three different directions ($0^{\circ }$, $-30^{\circ }$ and $+30^{\circ }$) are averaged, and an activity label associated with the maximum averaged output is selected.

Classifier performance is evaluated using accuracy, defined as the rate of correct predictions, and the F1-score is averaged across all activities. Table 3 reports the results of experiments conducted under the large labeled data setting $E_{1}V_{1}T_{62}$ using three beamforming directions. The results indicate that the classifiers achieve strong performance when sufficient labeled data are available. In addition, the classifier for Node2 performs best, followed by the classifier for Node3.

Table 4 shows the results of experiments conducted under the large labeled data setting $E_{1}V_{1}T_{62}$ using a single beamforming direction ($0^{\circ }$). As observed previously, Node2 achieves the best performance, followed by Node3. However, the performance of both nodes degrades slightly compared with classifiers that use three beamforming directions. This result confirms that spacial information plays an important role in activity recognition performance.

Table 5 shows the results of experiments conducted under the small labeled data setting $T_{1}V_{1}E_{62}$ using three beamforming directions. In contrast to the large labeled data setting $E_{1}V_{1}T_{62}$, both evaluation metrics degrade substantially. Although each classifier operates within a relatively constrained hypothesis space defined by 128-dimensional feature representations, approximately 400 labeled samples are insufficient to train an accurate classifier under this setting.

Based on the experimental results obtained thus far, the remainder of this paper presents results using the microphone array node Node2.

### 3.5. Unsupervised Leaning

To investigate the feasibility of the unsupervised learning, we explore two clustering algorithms, *K*-Means and Spectral Clustering. For *K*-Means, the same feature vectors described in the previous section are used. Specifically, each feature vector is obtained by averaging 748 embedding vectors across all frames within an audio segment. In contrast, Spectral Clustering uses embedding vectors from all frames within each audio segment, following the approach described in [33]. In this case, each feature vector is constructed by horizontally concatenating the 748 embedding vectors of dimension 128 for each audio segment. While this approach has the clear advantage of preserving fine-grained acoustic characteristics with higher temporal resolution, it also introduces a risk of overfitting due to the resulting high-dimensional hypothesis space. Previous studies [32,33] have shown that subspace clustering methods are effective in such settings because of the low-rank subspaces within the high-dimensional space.

Given a predefined number of clusters *K*, both algorithms partition the entire dataset into *K* clusters without using activity labels. After clustering, each cluster is mapped to an activity label using majority voting, which serves as a proxy for querying users about which activity corresponds to each cluster. The majority voting procedure is defined as follows: for each cluster *c*, the ground truth activity labels of all data points belonging to that cluster are collected, and the most frequent label m(c) is assigned to the cluster. Data points in the evaluation dataset are excluded from this process. Although clustering is performed over all data points in the dataset, majority voting is applied only to data points in the training and validation datasets. Specifically, under the large labeled data setting $E_{1}V_{1}T_{62}$, data points from 63 folds are used, whereas under the small labeled data setting $T_{1}V_{1}E_{62}$, data points from two folds are used. For each data point in the evaluation dataset, if the cluster *c* to which it belongs has an assigned activity label m(c), that label is used. Otherwise, the ‘other’ activity is assigned to the data point. Based on the predicted activities for all evaluation data points, accuracy and F1-score are computed.

**Figure 2:**
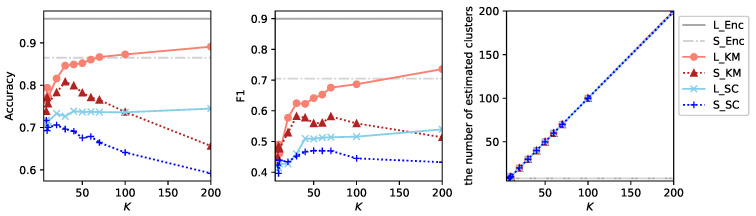
Results of experiments using *K*-Means (L_KM and S_KM) and Spectral Clustering (L_SC and S_SC) applied to a single audio stream enhanced toward direction $0^{\circ }$. For different values of *K*, accuracy, F1-score and the number of estimated clusters are shown. L_KM and L_SC denote results under the large labeled data setting $E_{1}V_{1}T_{62}$, whereas S_KM and S_SC denote results under the small labeled data setting $T_{1}V_{1}E_{62}$. For reference, results from supervised learning using three audio streams are also included (L_Enc for $E_{1}V_{1}T_{62}$ and S_Enc for $T_{1}V_{1}E_{62}$).

Figure 2 shows the results of experiments using clustering algorithms applied to a single audio stream enhanced toward the direction $0^{\circ }$, where we used sklearn.cluster.KMeans and sklearn.cluster.SpectralClustering ( parameters are set as affinity=‘nearest_neighbor’ and assign_labels=‘discretize’ to obtain the best results) implementations from the Scikit-learn [44] toolbox. This result indicates that Spectral Clustering performs worse than *K*-Means under this configuration. We hypothesize that this performance degradation is attributed to the excessively high-dimensional hypothesis space. To examine this possibility, we additionally apply Spectral Clustering to feature vectors obtained by averaging the embedding vectors for each audio segment.

**Figure 3:**
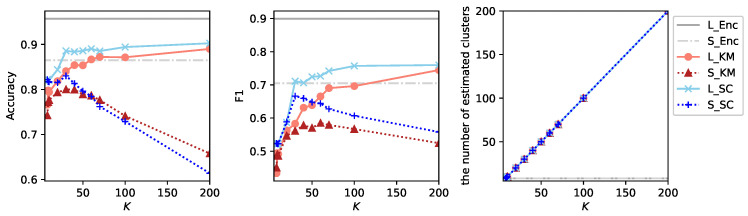
Results of experiments using *K*-Means (L_KM and S_KM) and Spectral Clustering (L_SC and S_SC) with three audio streams enhanced toward the directions $0^{\circ }$, $-30^{\circ }$ and $+30^{\circ }$. For different values *K*, accuracy, F1-score and the number of estimated clusters are shown.

Regarding the additional experiment with Spectral Clustering, we report the results obtained using three audio streams, as *K*-Means with three audio streams performs slightly better than its single-stream counterpart. For each audio segment with three audio streams, three feature vectors are obtained by averaging the embedding vectors across all frames within each stream. These vectors are concatenated to form a single feature vector. Figure 3 shows the results of clustering algorithms under this configuration.

In this configuration, Spectral Clustering performs better than *K*-Means particularly with respect to the F1-score. However, under the small labeled data setting $T_{1}V_{1}E_{62}$, the performance of both S_KM and S_SC degrades rapidly once *K* exceeds a certain threshold. Notably, this phenomenon does not occur under the large labeled data setting $E_{1}V_{1}T_{62}$ even though clustering is performed on the entire dataset and identical clusters are therefore estimated in both settings. This behavior suggests that, for large values of *K*, many data points in the evaluation dataset of $T_{1}V_{1}E_{62}$ do not belong to clusters that are well represented in the training and validation datasets for a large *K*. As *K* increases, the number of estimated clusters grows almost linearly for both algorithms, which is the expected behavior. These observations indicate that most newly created clusters at a large *K* are not shared between labeled and unlabeled data. In other words, sufficient generalization has not been achieved, and the clustering process exhibits characteristics of rote learning rather than transferable structure learning.

For a smaller values of *K*, around 25, a certain degree of generalization is observed, along with several local peaks in both evaluation metrics. However, to exploit this behavior effectively, a principled procedure is required to determine an appropriate value of *K*.

Given these limitations, we turn to a more structured unsupervised learning approach based on DMM models.

## 4. The DMM Model for Clustering of Audio Segments

This paper considers a generative probabilistic model that generates neural audio codec codes conditioned on latent activities. This formulation is conceptually similar to the ATM, which generates acoustic words conditioned on latent acoustic topics. While ATMs typically rely on LDA models, the authors have shown [9] that for signals exhibiting the burstiness property, DMM models are more suitable for modeling the underlying generative process. In this section, after demonstrating that codes obtained from the neural audio codec also exhibit burstiness, we extend the DMM model to generate multiple streams of codes. We then present a Gibbs sampler  [13] for estimating the parameters of the extended model.

### 4.1. Acoustic Words

The neural audio codec EnCodec employed in this paper uses a residual vector quantizer (RVQ) as its bottleneck representation. Each embedding vector obtained from the final layer of the encoder is first projected onto the closest entry in a codebook, and the corresponding residual is computed. This residual is subsequently projected onto the closest entry in a second codebook, and a new residual is again computed. By repeating this process, a sequence of codes representing the input embedding is obtained. RVQ is designed to control the accuracy of encoding based on the number of quantization stages. Increasing the number of quantization stages, that is, increasing the number of codes per frame, allows the input audio frame to be encoded with higher precision. By default, a sequence of eight codes encodes each audio frame, but the proposed method uses only the first two codes as an acoustic word. Specifically, an acoustic word is constructed by concatenating these first two codes. This corresponds to performing an approximation of each input audio frame that is even coarser than RVQ. While this coarse approximation reduces computational complexity, the working hypothesis is that it provides sufficient approximation to distinguish activities in activity recognition, unlike in speech recognition.

### 4.2. Generative Probabilistic Models for Acoustic Words

When each acoustic word $w_{n}\in \left\{1,\ldots ,V\right\}$ (1≤n≤N) is assumed to follow a categorical distribution parameterized by $\phi =\phi_{1},\ldots ,\phi_{V}$ ($\sum_{v}^{V}\phi_{v}=1$), the probability of generating an observation vector $w=w_{1},\ldots ,w_{N}$ can be expressed as

$$P\left(w|\phi \right)=\prod_{n}^{N}\prod_{v}^{V}\phi_{v}^{\left[w_{n}=v\right]}=\prod_{v}^{V}\phi_{v}^{x_{v}},$$

where $x_{v}=\sum_{n}^{N}\left[w_{n}=v\right]$ and [·] denotes the Iverson bracket, i.e., [P]=1 if *P* is true and [P]=0 otherwise.

For any count vector $x=(x_{1},\ldots ,x_{V})$, x then follows a multinomial distribution parameterized by ϕ:

$$\begin{array}{c} \mathrm{Mul}\left(x|\phi \right)=N!\prod_{v}^{V}\frac{\phi_{v}^{x_{v}}}{x_{v}!}, \end{array}$$

where $N=\sum_{v}^{V}x_{v}$. A Dirichlet multinomial (DM) distribution is obtained as a compound distribution by marginalizing over all possible multinomial distributions [45]. As the conjugate prior of the multinomial distribution $\mathrm{Mul}\left(x|\phi \right)$, the Dirichlet distribution $\mathrm{Dir}\left(\phi |\beta \right)$ is employed. The resulting DM distribution is given by

(1) $$\begin{array}{cc} \mathrm{DM}\left(x|\beta \right) & =\int_{\phi }\mathrm{Dir}\left(\phi |\beta \right)\mathrm{Mul}\left(x|\phi \right)d\phi \\ \; & =\frac{\Gamma \left(N+1\right)\Gamma \left(\beta_{\cdot }\right)}{\Gamma \left(\beta_{\cdot }+N\right)}\prod_{v}^{V}\frac{\Gamma \left(x_{v}+\beta_{v}\right)}{\Gamma \left(x_{v}+1\right)\Gamma \left(\beta_{v}\right)}, \end{array}$$

where $\beta_{\cdot }=\sum_{v}^{V}\beta_{v}$.

### 4.3. Burstiness

**Figure 4:**
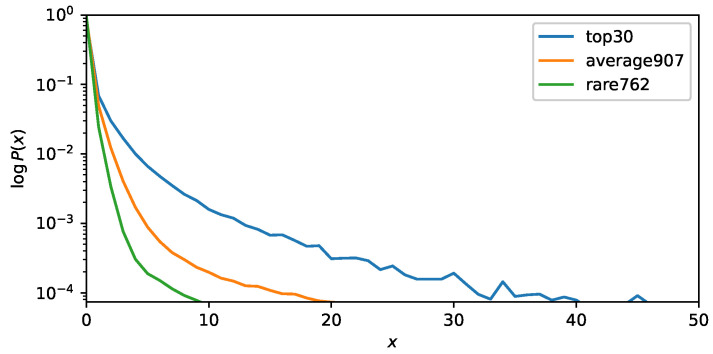
Power-law behavior of count probabilities for acoustic words in the dataset used in the experiment described in Section 5.1. The probabilities of words appearing exactly *x* times within an activity are averaged over three groups: the 30 most frequently occurring words, 907 words with approximately average frequency, and 762 rarely occurring words.

Figure 4 shows the count probabilities of observed acoustic words obtained from audio signals enhanced toward the direction $0^{\circ }$, which are used in the experiment described in Section 5.1. All 1699 acoustic words are divided into three groups based on their observed frequency: the 30 most frequently occurring words, 907 words with approximately average frequency, and 762 rarely occurring words. For each word, the probability of appearing exactly *x* times within a given activity is computed and then averaged within each group. If *x* follows a multinomial distribution, the probability of observing exactly *x* occurrences decays exponentially and appears as a straight descending line on a semi-logarithmic plot. In practice, however, the count probabilities of all three-word groups follow a power-law distribution. This behavior can be attributed to burstiness, that is, once an acoustic word appears during an activity, it tends to occur repeatedly within that activity even when its overall frequency across the dataset is low.

The DM distribution is capable of capturing this power-law behavior. By fixing $x_{u}\left(u\neq v\right)$ as constants, (1) can be rewritten as $P\left(x_{v}\right)=C\frac{\Gamma \left(x_{v}+N^{\prime }+1\right)\Gamma \left(x_{v}+\beta_{v}\right)}{\Gamma \left(x_{v}+N^{\prime }+\beta_{\cdot }\right)\Gamma \left(x_{v}+1\right)}$, where $N^{\prime }=N-x_{v}$ and *C* is a constant. For sufficiently large $x_{v}$, $P(x_{v})$ can be approximated as $O(x_{v}^{\beta_{v}-\beta_{\cdot }})$ [9]. These results indicate that the DM distribution is well suited for modeling the generation process of acoustic words obtained from the neural audio codec.

### 4.4. DMM Models

For any observation vector w, the DM distribution is written as follows without considering the permutation of the individual counts $x_{v}$:

(2) $$\begin{array}{c} \mathrm{DM}\left(w|\beta \right)=\frac{\Gamma \left(\beta_{\cdot }\right)}{\Gamma \left(\beta_{\cdot }+N\right)}\prod_{v}^{V}\frac{\Gamma \left(x_{v}+\beta_{v}\right)}{\Gamma \left(\beta_{v}\right)}=\frac{B\left(x+\beta \right)}{B\left(\beta \right)}, \end{array}$$

where B(·) denotes the multi-variate beta function, defined as $B\left(\beta \right)=\frac{\prod_{v}^{V}\Gamma \left(\beta_{v}\right)}{\Gamma \left(\beta_{\cdot }\right)}$.

It is natural to assume that the generative distribution depends on latent activities and that each observation is generated through a mixture process over activities:

$$\begin{array}{ccc} P(w) & = & \sum_{k}^{K}P\left(z=k\right)P\left(w|z=k\right), \end{array}$$

where *z* is a random variable representing one of *K* latent activities. Following Nigam et al. [10], the mixture weight P(z=k) is assumed to follow a categorical distribution parameterized by θ, and θ itself follows a Dirichlet distribution parameterized by $\alpha =(\alpha_{1},\ldots ,\alpha_{K})$, as given below.

$$P\left(w|\alpha ,\beta \right)=\int_{\theta }\mathrm{Dir}\left(\theta |\alpha \right)\;\sum_{k}^{K}P\left(z=k|\theta \right)\mathrm{DM}\left(w|z=k,\beta_{k}\right)d\theta .$$

This mixture formulation is referred to as the DMM model [11].

**Figure 5:**
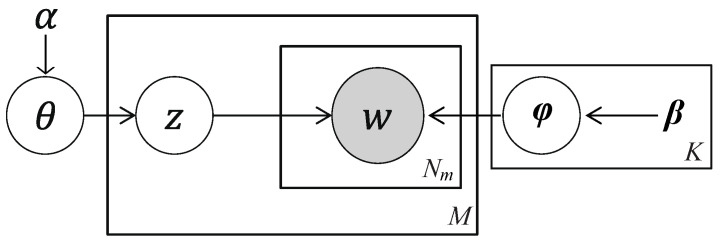
The graphical model for the DMM model.

Figure 5 illustrates the graphical model representation of the DMM model.

Given observation $W=w_{1},\ldots ,w_{M}$ and unobservable latent activities $Z=z_{1},\ldots ,z_{M}$, where $z_{m}\in \left\{1,\ldots ,K\right\}$ and $w_{m}=w_{m,1},\ldots ,w_{m,N_{m}}$ (1≤m≤M) denotes the sequence of words in the *m*-th observation with length $N_{m}$, the joint probability decomposes into two factors:

$$\begin{array}{c} P(W,Z|\beta ,\alpha )=P(Z|\alpha )P(W|Z,\beta ). \end{array}$$

The first factor is given by

(3) $$\begin{array}{ccc} P(Z|\alpha ) & = & \int_{\theta }\mathrm{Dir}\left(\theta |\alpha \right)\prod_{k}^{K}{\left(\theta_{k}\right)}^{\tau_{k}}\;d\theta \\ & = & \frac{B\left(\tau +\alpha \right)}{B\left(\alpha \right)}, \end{array}$$

where $\tau =(\tau_{1},\ldots ,\tau_{K})$ denotes activity count vectors with $\tau_{k}=\sum_{m}^{M}\left[z_{m}=k\right]$. The second factor is given by

(4) $$\begin{array}{ccc} P(W|Z,\beta ) & = & \prod_{k}^{K}\int_{\phi_{k}}\mathrm{Dir}\left(\phi_{k}|\beta_{k}\right)\prod_{v}^{V}\phi_{k,v}^{\omega_{k,v}}\;d\phi_{k}\\ & = & \prod_{k}^{K}\frac{B\left(\omega_{k}+\beta_{k}\right)}{B\left(\beta_{k}\right)}, \end{array}$$

where $\omega_{k}=\left(\omega_{k,1},\ldots ,\omega_{k,V}\right)$ represents the word count vector for activity *k*, defined as $\omega_{k,v}=\sum_{m}^{M}\sum_{n}^{N_{m}}\left[z_{m}=k\;and\;w_{m,n}=v\right]$ for each *k* (1≤k≤K) and *v* (1≤v≤V).

### 4.5. Extension to Multiple Streams of Acoustic Words

To accommodate multiple sound directions, we extend the DMM model to emit multiple streams of acoustic words. Without loss of generality, we consider a DMM model formulation with two output streams.

**Figure 6:**
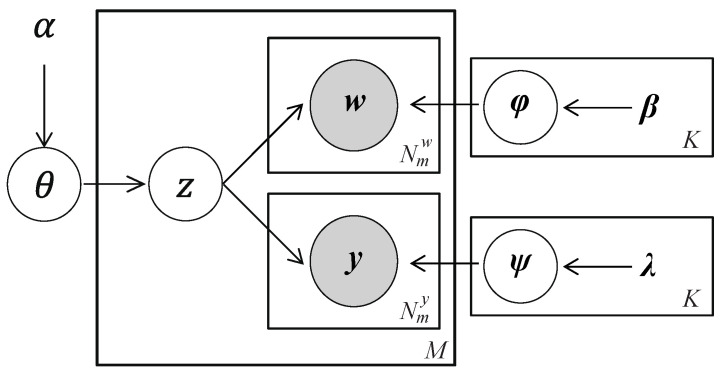
The graphical model representation of the DMM model with two streams of acoustic words.

Given two streams of observation $W=w_{1},\ldots ,w_{M}$ and $Y=y_{1},\ldots ,y_{M}$, where $w_{m}=w_{m,1},\ldots ,w_{m,N_{m}^{w}}$ (1≤m≤M) represents a sequence of words of length $N_{m}^{w}$ in one stream and $y_{m}=y_{m,1},\ldots ,y_{m,N_{m}^{y}}$ (1≤m≤M) represents a sequence of words of length $N_{m}^{y}$ in another stream, the joint probability is defined as

$$\begin{array}{c} P(W,Y,Z|\alpha ,\beta ,\lambda )=P(Z|\alpha )P(W|Z,\beta )P(Y|Z,\lambda ), \end{array}$$

where λ denotes the hyper-parameters of the Dirichlet distributions for the second stream. Here, it is assumed that $w_{m}$ and $y_{m}$ are conditionally independent given the latent activity assignment $z_{m}$. Figure 6 illustrates the graphical model representation of the DMM model with two streams of acoustic words.

Under this model, a Gibbs sampler for estimating the unobserved activity assignment Z within the MCMC method is derived as follows.

(5) $$\begin{array}{c} P\left(z_{m}=k|Z^{\setminus m},W,Y,\alpha ,\beta ,\lambda \right)=\frac{P(Z,W,Y|\alpha ,\beta ,\lambda )}{P(Z^{\setminus m},W,Y|\alpha ,\beta ,\lambda )} \end{array}$$

(6) $$\begin{array}{ccc} & = & \frac{P(Z|\alpha )P(W,Y|Z,\beta ,\lambda )}{P\left(Z^{\setminus m}|\alpha \right)P\left(w_{m},W^{\setminus m},y_{m},Y^{\setminus m}|Z^{\setminus m},\beta ,\lambda \right)} \end{array}$$

(7) $$\begin{array}{ccc} & = & \frac{P(Z|\alpha )P(W|Z,\beta )P(Y|Z,\lambda )}{P\left(Z^{\setminus m}|\alpha \right)P\left(W^{\setminus m}|Z^{\setminus m},\beta \right)P\left(w_{m}|Z^{\setminus m},\beta \right)P\left(Y^{\setminus m}|Z^{\setminus m},\lambda \right)P\left(y_{m}|Z^{\setminus m},\lambda \right)} \end{array}$$

(8) $$\begin{array}{ccc} & \propto & \frac{P(Z|\alpha )}{P(Z^{\setminus m}|\alpha )}\frac{P(W|Z,\beta )}{P(W^{\setminus m}|Z^{\setminus m},\beta )}\frac{P(Y|Z,\lambda )}{P(Y^{\setminus m}|Z^{\setminus m},\lambda )}, \end{array}$$

where $Z^{\setminus m}=z_{1},\ldots ,z_{m-1},z_{m+1},\ldots ,z_{M}$, $W^{\setminus m}=w_{1},\ldots ,w_{m-1},w_{m+1},\ldots ,w_{M}$, $Y^{\setminus m}=y_{1},\ldots ,y_{m-1},y_{m+1},\ldots ,y_{M}$, and to obtain (7), it is assumed that $w_{m}$ and $w_{m^{\prime }}$ ($m^{\prime }\neq m$) are conditionally independent given $z_{m}$, and so are $y_{m}$ and $y_{m^{\prime }}$.

Following the same reasoning as in prior work [9], each factor can be computed analytically as follows.

(9) $$\begin{array}{c} \frac{P(Z|\alpha )}{P(Z^{\setminus m}|\alpha )}=\frac{\tau_{k}^{\setminus m}+\alpha_{k}}{M-1+\alpha_{\cdot }}, \end{array}$$

where $\alpha_{\cdot }=\sum_{k}^{K}\alpha_{k}$ and $\tau_{k}^{\setminus m}=\tau_{k}-\left[z_{m}=k\right]$.

(10) $$\begin{array}{ccc} \frac{P(W|Z,\beta )}{P(W^{\setminus m}|Z^{\setminus m},\beta )} & = & \frac{\prod_{v}^{V}\prod_{\ell =1}^{N_{m}^{w}\left(v\right)}\left(\omega_{k,v}^{\setminus m}+\beta_{k,v}+\ell -1\right)}{\prod_{n=1}^{N_{m}^{w}}\left(\omega_{k,\cdot }^{\setminus m}+\beta_{k,\cdot }+n-1\right)} \end{array}$$

(11) $$\begin{array}{ccc} \frac{P(Y|Z,\lambda )}{P(Y^{\setminus m}|Z^{\setminus m},\lambda )} & = & \frac{\prod_{u}^{U}\prod_{\ell =1}^{N_{m}^{y}\left(u\right)}\left(\eta_{k,u}^{\setminus m}+\lambda_{k,u}+\ell -1\right)}{\prod_{n=1}^{N_{m}^{y}}\left(\eta_{k,\cdot }^{\setminus m}+\lambda_{k,\cdot }+n-1\right)} \end{array}$$

where *U* is the number of words for the stream Y, $\eta_{k,u}=\sum_{m}^{M}\sum_{n}^{N_{m}^{y}}\left[z_{m}=k\;and\;y_{m,n}=u\right]$, $\beta_{k,\cdot }=\sum_{v}^{V}\beta_{k,v}$, $\lambda_{k,\cdot }=\sum_{u}^{U}\lambda_{k,u}$, $\omega_{k,\cdot }=\sum_{v}^{V}\omega_{k,v}$, $\eta_{k,\cdot }=\sum_{u}^{U}\eta_{k,u}$, $N_{m}^{w}\left(v\right)=\sum_{n}^{N_{m}^{w}}\left[w_{m,n}=v\right]$, $N_{m}^{y}\left(u\right)=\sum_{n}^{N_{m}^{y}}\left[y_{m,n}=u\right]$, $\omega_{k,v}^{\setminus m}=\omega_{k,v}-N_{m}^{w}\left(v\right)$, $\eta_{k,u}^{\setminus m}=\eta_{k,u}-N_{m}^{y}\left(u\right)$, $\omega_{k,\cdot }^{\setminus m}=\sum_{v}^{V}\omega_{k,v}^{\setminus m}$, and $\eta_{k,\cdot }^{\setminus m}=\sum_{u}^{U}\eta_{k,u}^{\setminus m}$.

According to (9)–(11) the time complexity required for the Gibbs sampling is O(KLS), where *L* is the number of streams and *S* the maximum number of observations in any segment of any stream. The space complexity is O(KLVM), where *V* is the maximum number of vocabularies of any stream.

Notably, streams W and Y may have different vocabularies and different sequence length, $N_{m}^{w}$ and $N_{m}^{y}$, for each observation *m*. Consequently, each stream can correspond to a different sensing modality. This flexibility allows the expanded DMM model to support heterogeneous sensor networks. Moreover, the formulation naturally extends to a variant of the DMM model that captures Markov dependencies between activities [9].

## 5. Experimental Results

As discussed above, the activity ‘absence’ is excluded because it can be detected relatively easily using other sensors such as PIR motion sensors. Furthermore, acoustic words appearing in the activity ‘absence’ are also filtered, under the assumption that they originate from silence or environmental sounds unrelated to daily activities. In addition, to improve computational efficiency and numerical stability, acoustic words are filtered based on their frequency of occurrence. Given a term frequency ratio (TFR) *r*, words are sorted in descending order of frequency. Words are retained until the cumulative frequency relative to the total frequency exceeds *r*, and then the remaining words are discarded. In the experiments reported below, *r* is initially set to 0.6, while Section 5.2 examines other values to evaluate sensitivity to this parameter.

**Figure 7:**
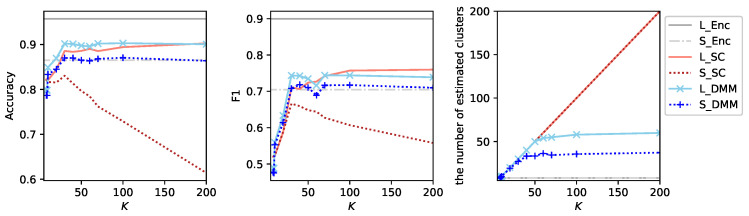
Results of experiments using DMM models with three streams of acoustic words: L_DMM denotes results under the large labeled data setting $E_{1}V_{1}T_{62}$, whereas S_DMM denotes results under the small labeled data setting $T_{1}V_{1}E_{62}$. For reference, the results shown in Figure 3 are also included.

Figure 7 summarizes the performance of the DMM models with three streams of acoustic words. As *K* increases, the number of estimated clusters converges to nearly constant values: approximately 50 under $E_{1}V_{1}T_{62}$ and approximately 30 under $T_{1}V_{1}E_{62}$. Under the small labeled data setting $T_{1}V_{1}E_{62}$, once *K* exceeds 30, both accuracy and F1-score for S_DMM reach levels comparable to those achieved by supervised learning S_Enc. Because the number of estimated clusters stabilizes at 30, querying users about roughly 30 clusters allow the DMM model to achieve performance comparable to S_Enc, which relies on approximately 400 labeled samples. Moreover, if additional data points become available during the inquiry process, the DMM model can surpass the performance of S_Enc using the same number of queries, as indicated by the L_DMM results.

Finally, it is notable that no explicit procedure for selecting an optimal value of *K* seems to be required. When *K* is chosen sufficiently large, the effective number of clusters is determined automatically based on intrinsic complexity of the data. It is conceivable that the strong model bias of the DMM model, which places greater emphasis on words with bursty occurrences, helps prevent overfitting as the model capacity increases.

### 5.1. Effectiveness of Multiple Streams of Acoustic Words

**Figure 8:**
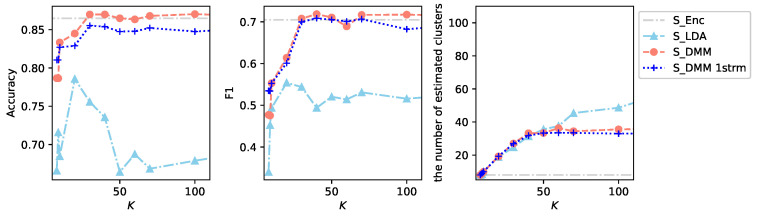
Results of experiments using DMM models with a single stream of acoustic words: S_DMM 1strm denotes the DMM model using a single stream obtained from the beam direction $0^{\circ }$ under the small labeled data setting $T_{1}V_{1}E_{62}$. S_LDA denotes the results of the LDA model under the same conditions. For reference, the results shown in Figure 7 are also included.

As an ablation study, an experiment is conducted using the DMM model with a single stream of acoustic words obtained from audio signals enhanced toward the direction $0^{\circ }$. Figure 8 presents the result of the single-stream DMM model under the small labeled data setting $T_{1}V_{1}E_{62}$. When the two additional streams corresponding to the $-30^{\circ }$ and $+30^{\circ }$ directions are not used, both accuracy and F1-score show a slight degradation. The number of estimated clusters is also marginally reduced, reflecting decreased lexical diversity in the acoustic word representation. These results indicate that incorporating multiple streams of acoustic words contributes positively to clustering quality and overall recognition performance, thereby confirming the effectiveness of the multi-stream extension of the DMM model.

Figure 8 also presents the results of the LDA model obtained under the same experimental conditions. The LDA model and its variants are used in ATMs. In the ATMs, topics are estimated for each individual acoustic word in a segment. Therefore, an additional procedure is required to determine the topic of the segment. In the present study, the performance is evaluated by assigning the topic with highest frequency within the segment. We used the class models.ldamodel in the Gensim toolbox [46]. For the parameter num_topics, different numbers *K* of topics were attempted, and the parameters alpha and eta were set to auto, which indicates that asymmetric values are learned from the data. All other parameters were set to their default values.

Since the LDA model accepts only a single stream of words, it is compared with S_DMM 1strm. The results show that the DMM model outperforms the LDA model for the data of acoustic words exhibiting the burstiness, just as it did for the data of PIR motion sensors exhibiting a similar burstiness property [9].

### 5.2. Effects of Filtering Acoustic Words

To analyze sensitivity to the TFR, experiments are conducted using multiple TFR thresholds. In addition to the TFR value of 0.6 used in the preceding experiments, thresholds of 0.2, 0.3, 0.4 and 0.5 are applied to filter acoustic words from each beamforming direction. Table 6 summarizes the resulting vocabulary sizes for each TFR and beamforming direction.

**Figure 9:**
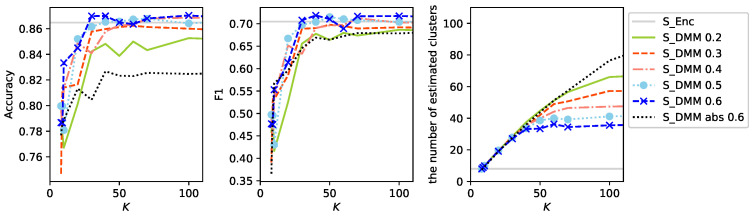
Results of experiments for different term frequency ratios (TFRs): S_DMM *r* denotes results under the small labeled data setting $T_{1}V_{1}E_{62}$ using the DMM model with three streams of acoustic words filtered by the TFR threshold *r*. S_DMM abs 0.6 is the same as S_DMM 0.6 but shows the result when words appearing in the activity ‘absence’ are not removed.

Figure 9 presents the experimental results obtained for different TFR values. Overall, large vocabulary size is associated with improved performance. However, performance differences become negligible once the TFR reaches 0.4 or higher. When the TFR exceeds 0.6, both accuracy and F1-score tend to become unstable. This behavior is likely attributable to numerical instability arising from the rapid growth of the denominator in Equation (10), although further investigation is required to confirm this hypothesis. It is also notable that increasing the TFR leads to a reduction in the number of estimated clusters. One plausible explanation is that as the denominator grows rapidly, the influence of relatively low-frequency acoustic words is diminished, resulting in coarser clustering behavior.

In the figure, S_DMM abs 0.6 shows the result when acoustic words appearing in the activity ‘absence’ are not removed and only the filtering with TFR 0.6 is applied. After the filtering, streams corresponding to the $0^{\circ }$, $-30^{\circ }$, and $+30^{\circ }$ directions contain 310, 298, and 289 words, respectively. Compared with S_DMM 0.6, the numbers of remaining words are substantially reduced even though filtering with the same TFR threshold is applied for larger vocabularies. This indicates that some words appearing in the activity ‘absence’ also occur with exceptionally high frequency in the other activities. Furthermore, performance degradation is observed not only relative to S_DMM 0.6 but also when compared to S_DMM 0.3, which uses vocabularies with similar sizes. These results suggest that eliminating words originating from the activity ‘absence’ effectively removes noisy words unrelated to activities being predicted.

## 6. Limitations and Future Work

Although the effectiveness of the DMM models for activity recognition from daily-life sounds has been demonstrated using the DCASE2018 Challenge Task5 dataset, it remains necessary to verify whether the proposed approach generalizes to other datasets. In particular, the dataset consists of recordings from a young male; therefore, it remains unclear whether comparable performance can be achieved for daily-life sounds produced by elderly individuals. In practice, age-related declines in physical functions may cause daily movements to become slower or weaker, potentially leading to changes in the acoustic characteristics of everyday sounds. Additionally, compensating for age-related deterioration in auditory function by increasing the volume of a television or other audio sources may heighten susceptibility to background noise. The proposed method aims to learn an individualized model from scratch through unsupervised learning; therefore, it is expected to adapt to user-specific characteristics without assuming models trained on data from younger individuals or other users. However, it remains necessary to examine whether performance comparable to that demonstrated in this study can be achieved when daily sounds become weaker and are observed under increased noise conditions. To the best of the authors’ knowledge, publicly available datasets capturing daily-life sounds of older adults are extremely scarce, with “The Sounds of Home” dataset [47] being one of the very few datasets available, but for acoustic event detection. Consequently, determining the true effectiveness of the proposed method for activity recognition based on the daily-life sounds of older adults remains a subject for future work, including the acquisition of appropriate data.

Another limitation of the DCASE2018 Task5 dataset is that it contains only audio segments fully contained within a single activity, excluding segments spanning multiple activities. To demonstrate effectiveness under more realistic conditions, it is necessary to evaluate performance on continuous audio streams that include activity transitions. The SINS dataset [37], from which the DCASE dataset was derived, includes week-long continuous recordings and may therefore be suitable for such validation. When applying the proposed approach to continuous recordings, DMM models incorporating Markov dependencies between activities [9] may be effective as consecutive audio segments typically exhibit temporal continuity.

In the present study, no convergence analysis for Gibbs sampling was performed. Instead, the number of iterations of Gibbs sampling was set to 50 for both training and inference, and visual inspection was performed to determine whether the parameters appeared stable or not. Convergence assessment is important future work for developing a practical system.

The overall time complexity of the proposed clustering algorithm scales linearly with the number of iterations of Gibbs sampling, number of clusters, number of audio segments, number of streams, and maximum number of acoustic words for any segment in any stream. Actual execution time was 393 s for clustering 13,531 audio segments and 0.13 s for estimating a cluster of a 10 s audio segment on an AMD Ryzen 9 5950X 2.2 GHz CPU. These times do not include the time required for encoding, the number of clusters was set to 50, and the threshold TFR was set to 0.6. Based on these facts, cloud execution seems feasible, though execution on edge devices might be difficult. Exploring a more efficient implementation is also future work.

In the present study, signal processing performed prior to applying the neural audio codec was not explored sufficiently. While classical DAS beamforming employed in this study may not achieve precise sound source separation and the signal may be somewhat distorted, we expect it to improve clustering performance by leveraging the spatial information manifested as differences in the encodings of each stream, dependent on whether the sound is directional or omni-directional. If the cost of adapting to individual living environments is acceptable, we would also like to explore noise suppression originating from a television using more elaborate sound source separation techniques.

Regarding noise reduction after encoding by a neural audio codec, noise can in principle be suppressed simply by removing noise-related acoustic words. However, in unsupervised learning settings, the target daily activities to be recognized are not predetermined, making it generally difficult to define what constitutes noise in advance. In this study, words appearing in the activity ‘absence’ were treated as noise, under the assumption that they originate from silence or environmental sounds unrelated to daily activities, and were removed accordingly. The experiment described in Section 5.2 demonstrated the effectiveness of this approach. Moreover, although not addressed in this study, we consider that the strong model bias of the DMM model, designed to capture burstiness, may also contribute to noise reduction. For example, noise-related words, such as those generated by accidentally dropping an object during an activity, are unlikely to appear repeatedly, whereas activity-related words exhibit bursty occurrences. Thus, the DMM model, which inherently favors words with bursty occurrences, may suppress the influence of noise-related words. Further analysis in this direction represents an interesting avenue for future research.

Section 5.2 also discusses the impact of word filtering using the TFR threshold. As the vocabulary size increases, the computational resources required for both training and inference grow substantially, and the risk of unstable computation results also increases. Therefore, it is desirable to keep the vocabulary size as small as possible. However, as the experimental results indicate, too small a vocabulary naturally leads to the loss of acoustic words necessary for distinguishing activities, which in turn degrades clustering performance. It was also observed that performance gradually saturates even when the vocabulary size is increased. During this process, the estimated number of clusters consistently decreases, suggesting that unnecessary clusters are not being generated by added noise-derived vocabulary. Elucidating these phenomena and deriving guidelines for setting the TFR threshold remain important tasks for future work. In addition to this system parameter, several other system-level design choices that affect performance must be made in order to develop a practical system, such as a design of input streams or the appropriate number of codebooks to produce acoustic words.

Heterogeneous sensing modalities can complement one another. For example, PIR motion sensors impose minimal privacy intrusion and have low installation and maintenance costs, but they cannot distinguish between different activities occurring in the same location. In contrast, acoustic sensors are capable of differentiating activities that take place in the same area, though they cannot recognize actions that generate only faint sounds, and they are susceptible to noise as well as higher installation and maintenance costs. To use such sensors in a complementary manner, algorithms that can analyze data obtained from heterogeneous sensor networks are required. The multi-stream extension of the DMM model may also be effective for heterogeneous sensor networks. Because each stream maintains its own vocabulary and the number of words within a segment may vary across streams, the model can simultaneously analyze different sensing modalities, such as acoustic streams and PIR sensor signals. Applying the proposed model to the heterogeneous sensor networks is an important future work.

Another direction for future research concerns the design of inquiry strategies for activity labeling. To minimize user burden while maximizing recognition accuracy, it is necessary to develop a principled strategy for selecting which clusters to query. These strategies may prioritize clusters corresponding to long-duration activities or those occurring consistently at specific times or on recurring dates. Inquiry strategies should also support appropriate visualizations or prompt users to report their current activity when clusters are estimated.

In determining inquiry strategies, the ontology of clusters is expected to play a key role. By exploiting the hierarchical structure that naturally emerges from clustering, it may be possible to reduce the number of required queries and control the sequence of inquiries.

## 7. Conclusions

Motivated by the need to reduce the cost of developing activity-recognition models, we explored an unsupervised approach to recognizing daily activities from household sounds to support ambient assisted living for the elderly living alone. We focused on extending the Dirichlet multinomial mixture (DMM) model, which we previously demonstrated to be effective for PIR motion sensor data. Its effectiveness partly stems from its ability to capture the burstiness property; once a signal is observed during an activity, it tends to recur multiple times within that activity.

We showed that, when acoustic signals are discretized using the neural audio codec EnCodec [41], the resulting symbol distributions also exhibit burstiness. Encouraged by this observation, we extended the DMM model to incorporate spatial information by handling multiple streams of acoustic codes corresponding to different sound directions. For the extended model, a Gibbs sampler for estimating its parameters was also presented. By using a publicly available real-life dataset of household sounds, we demonstrated that the proposed model can form more accurate clusters than existing unsupervised clustering methods.

Through light weight user feedback about activities for obtained clusters, an activity recognition system can gradually improve its accuracy during use. We believe that the unsupervised clustering method could be a key component necessary for realizing an activity recognition system, which is deployable ‘as-is’ and is semi-automatically adaptable to individuals living in diverse home environments.

## Figures and Tables

**Table 1:** Statistics on the number of labeled samples under the large labeled data setting $E_{1}V_{1}T_{62}$ for Node2: The second, third, and fourth columns report the average, minimum, and maximum number of labeled samples for each activity across training and validation datasets. The final row reports the corresponding statistics aggregated over all activities.

Activity	Ave.	Min.	Max.
other	507.0	506	508
cooking	1261.0	1260	1262
dishwashing	350.4	350	351
eating	568.0	567	569
social_activity	1216.7	1210	1220
vacuum_cleaner	239.2	238	240
watching_tv	4589.2	4586	4592
working	4588.2	4586	4590
all activities	13,319.6	13,304	13,328

**Table 2:** Statistics on the number of labeled samples under the small labeled data setting $T_{1}V_{1}E_{62}$ for Node2.

Activity	Ave.	Min.	Max.
other	16.1	15	17
cooking	40.0	38	42
dishwashing	11.1	10	12
eating	18.0	17	19
social_activity	38.6	32	52
vacuum_cleaner	7.6	7	9
watching_tv	145.7	141	152
working	145.7	143	149
all activities	422.8	410	451

**Table 3:** Results of experiments under the large labeled data setting $E_{1}V_{1}T_{62}$ for each microphone array node. Activity predictions are obtained by ensembling three audio streams enhanced toward the directions $0^{\circ }$, $-30^{\circ }$ and $+30^{\circ }$. The second column reports accuracy, defined as the rate of correct predictions, together with its standard deviation. The third column reports the F1-score averaged across all activities.

Node	Accuracy		F1-Score	
Node1	0.949	±0.016	0.876	±0.038
Node2	**0.957**	±0.015	**0.900**	±0.037
Node3	0.956	±0.014	0.894	±0.033
Node4	0.949	±0.017	0.875	±0.042

The largest value within the nodes is displayed in bold.

**Table 4:** Results of experiments under the large labeled data setting $E_{1}V_{1}T_{62}$ for each microphone array node. Activity predictions are obtained from a single audio stream enhanced toward the direction $0^{\circ }$.

Node	Accuracy		F1-Score	
Node1	0.934	±0.020	0.845	±0.048
Node2	**0.950**	±0.022	**0.882**	±0.050
Node3	0.944	±0.025	0.865	±0.058
Node4	0.940	±0.020	0.860	±0.046

The largest value within the nodes is displayed in bold.

**Table 5:** Results of experiments under the small labeled data setting $T_{1}V_{1}E_{62}$ for each microphone array node. Activity predictions are obtained by ensembling three audio streams enhanced toward the directions ($0^{\circ }$, $-30^{\circ }$ and $+30^{\circ }$ ).

Node	Accuracy		F1-Score	
Node1	0.849	±0.044	0.663	±0.112
Node2	0.865	±0.028	**0.705**	±0.064
Node3	**0.871**	±0.013	**0.705**	±0.031
Node4	0.848	±0.039	0.683	±0.034

The largest value within the nodes is displayed in bold.

**Table 6:** Vocabulary size for different term frequency ratios (TFRs). The first column lists the TFR threshold, and the final row reports the total number of acoustic words for each stream. The second, third and fourth columns correspond to the number of acoustic words obtained from the beamforming directions $0^{\circ }$, $-30^{\circ }$ and $+30^{\circ }$.

TFR	$0^{\circ }$	$-30^{\circ }$	$+30^{\circ }$
0.2	152	136	135
0.3	325	294	295
0.4	596	545	552
0.5	1007	925	942
0.6	1699	1559	1581
1.0	102,661	100,280	100,246

## Data Availability

The original data presented in the study are openly available at https://dcase.community/challenge2018/task-monitoring-domestic-activities-results (accessed on 25 February 2026). The software and processed data for reproducing the results of the study are available at https://github.com/sadohara/dmm2-for-asc (accessed on 25 February 2026).

## References

[1] The Cabinet Office of Japan (2024). Annual Report on the Aging Society: Summary FY2024. https://www8.cao.go.jp/kourei/english/annualreport/2024/pdf/2024.pdf.

[2] Mizuno J., Saito D., Sadohara K., Nihei M., Ohnaka S., Suzurikawa J., Inoue T. (2021). Effect of the Information Support Robot on the Daily Activity of Older People Living Alone in Actual Living Environment. Int. J. Environ. Res. Public Health.

[3] Mizuno J., Sadohara K., Nihei M., Onaka S., Nishiura Y., Inoue T. (2021). The application of an information support robot to reduce agitation in an older adult with Alzheimer’s disease living alone in a community dwelling: A case study. Hong Kong J. Occup. Ther..

[4] Cicirelli G., Marani R., Petitti A., Milella A., D’Orazio T. (2021). Ambient assisted living: A review of technologies, methodologies and future perspectives for healthy aging of population. Sensors.

[5] Cook D.J., Krishnan N. (2014). Mining the home environment. J. Intell. Inf. Syst..

[6] Amiribesheli M., Benmansour A., Bouchachia A. (2015). A Review of smart homes in healthcare. J. Ambient Intell. Humaniz. Comput..

[7] Leotta F., Mecella M., Sora D., Catarci T. (2019). Surveying human habit modeling and mining techniques in smart spaces. Future Internet.

[8] Hiremath S.K., Plötz T. (2023). The lifespan of human activity recognition systems for smart homes. Sensors.

[9] Sadohara K. (2022). Activity discovery using Dirichlet multinomial mixture models from discrete sensor data in smart homes. Pers. Ubiquitous Comput..

[10] Nigam K., McCallum A.K., Thrun S., Mitchell T. (2000). Text classification from labeled and unlabeled documents using EM. Mach. Learn..

[11] Jianhua Y., Wang J. (2014). A Dirichlet multinomial mixture model-based approach for short text clustering. Proceedings of the International Conference on Knowledge Discovery and Data Mining, New York, NY, USA, 24–27 August 2014.

[12] Kim S., Narayanan S.S., Sundaram S. (2009). Acoustic topic model for audio information retrieval. Proceedings of the 2019 IEEE Workshop on Applications of Signal Processing to Audio and Acoustics, WASPAA ’09, New Paltz, NY, USA, 18–21 October 2009.

[13] Griffiths T.L., Steyvers M. (2004). Finding scientific topics. Proc. Natl. Acad. Sci. USA.

[14] Cook D.J., Krishnan N.C., Rashidi P. (2013). Activity discovery and activity recognition: A new partnership. IEEE Trans. Cybern..

[15] Dimitrov T., Pauli J., Naroska E. (2010). Unsupervised recognition of ADLs. Artificial Intelligence: Theories, Models and Applications.

[16] Shang C., Chang C.Y., Chen G., Zhao S., Chen H. (2020). BIA: Behavior identification algorithm using unsupervised learning based on sensor data for home elderly. IEEE J. Biomed. Health Inform..

[17] Hoque E., Stankovic J. AALO: Activity recognition in smart homes using active learning in the presence of overlapped activities. Proceedings of the International Conference on Pervasive Computing Technologies for Healthcare.

[18] Saives J., Pianon C., Faraut G. (2015). Activity discovery and detection of behavioral deviations of an inhabitant from binary sensors. IEEE Trans. Autom. Sci. Eng..

[19] Leotta F., Mecella M., Sora D. (2020). Visual process maps: A visualization tool for discovering habits in smart homes. J. Ambient Intell. Humaniz. Comput..

[20] Barger T.S., Brown D.E., Alwan M. (2004). Health-status monitoring through analysis of behavioral patterns. IEEE Trans. Syst. Man Cybern. Part A Syst. Humans.

[21] Huỳnh T., Fritz M., Schiele B. (2008). Discovery of activity patterns using topic models. Proceedings of the International Conference on Ubiquitous Computing, Seoul, Republic of Korea, 21–24 September 2008.

[22] Niebles J.C., Wang H., Fei-Fei L. (2008). Unsupervised learning of human action categories using spatial-temporal words. Int. J. Comput. Vis..

[23] Sun F.T., Yeh Y.T., Cheng H.T., Kuo C., Griss M. Nonparametric discovery of human routines from sensor data. Proceedings of the International Conference on Pervasive Computing and Communications (PerCom).

[24] Tsai M.J., Wu C.L., Pradhan S.K., Xie Y., Li T.Y., Fu L.C., Zeng Y.C. (2016). Context-aware activity prediction using human behavior pattern in real smart home environments. Proceedings of the 2016 IEEE International Conference on Automation Science and Engineering (CASE), Fort Worth, TX, USA, 21–24 August 2016.

[25] Blei D., Ng A.Y., Jordan M.I. (2003). Latent Dirichlet allocation. J. Mach. Learn. Res..

[26] Teh Y.W., Jordan M.I., Beal M.J., Blei D.M. (2006). Hierarchical Dirichlet processes. J. Am. Stat. Assoc..

[27] Abeßer J. (2020). A review of deep learning based methods for acoustic scene classification. Appl. Sci..

[28] Igarashi A., Imoto K., Komatsu Y., Tsubaki S., Hario S., Komatsu T. How information on acoustic scenes and sound events mutually benefits event detection and scene classification tasks. Proceedings of the Asia-Pacific Signal and Information Processing Association Annual Summit and Conference (APSIPA ASC).

[29] Zhang H., Wu M., Cai X., Liu W. (2025). Scene-dependent sound event detection based on multitask learning with deformable large kernel attention convolution. PLoS ONE.

[30] Imoto K., Ohishi Y., Uematsu H., Ohmuro H. Acoustic scene analysis based on latent acoustic topic and event allocation. Proceedings of the International Workshop on Machine Learning for Signal Processing (MLSP).

[31] Imoto K., Ono N. (2019). Acoustic topic model for scene analysis with intermittently missing observations. IEEE/ACM Trans. Audio Speech Lang. Proc..

[32] Li S., Gu Y., Luo Y., Chambers J., Wang W. (2019). Enhanced streaming based subspace clustering applied to acoustic scene data clustering. Proceedings of the International Conference on Acoustics, Speech and Signal Processing (ICASSP), Brighton, UK, 12–17 May 2019.

[33] Wang M., Zhang X.L., Rahardja S. (2020). An unsupervised deep learning system for acoustic scene analysis. Appl. Sci..

[34] Liang Y., Chen W., Jiang A., Qiu Y., Zheng X., Huang W., Han B., Qian Y., Fan P., Zhang W.Q. Improving acoustic scene classification via self-supervised and semi-supervised learning with efficient audio transformer. Proceedings of the International Conference on Multimedia and Expo Workshops (ICMEW).

[35] Cai Y., Li S., Shao X. Leveraging self-supervised audio representations for data-efficient acoustic scene classification. Proceedings of the Workshop on Detection and Classification of Acoustic Scenes and Events.

[36] Dekkers G., Vuegen L., van Waterschoot T., Vanrumste B., Karsmakers P. (2018). DCASE 2018 Challenge—Task 5: Monitoring of Domestic Activities Based on Multi-Channel Acoustics.

[37] Dekkers G., Lauwereins S., Thoen B., Adhana M.W., Brouckxon H., van Waterschoot T., Vanrumste B., Verhelst M., Karsmakers P. The SINS database for detection of daily activities in a home environment using an acoustic sensor network. Proceedings of the Detection and Classification of Acoustic Scenes and Events 2017 Workshop (DCASE2017).

[38] Monitoring of Domestic Activities Based on Multi-Channel Acoustics (DCASE2018 Task5). https://dcase.community/challenge2018/task-monitoring-domestic-activities-results.

[39] Nakadai K., Okuno H.G., Mizumoto T. (2017). Development, deployment and applications of robot audition open source software HARK. J. Robot. Mechatron..

[40] Zeghidour N., Luebs A., Omran A., Skoglund J., Tagliasacchi M. (2021). SoundStream: An end-to-end neural audio codec. IEEE/ACM Trans. Audio Speech Lang. Proc..

[41] Défossez A., Copet J., Synnaeve G., Adi Y. (2022). High fidelity neural audio compression. arXiv.

[42] Turian J., Shier J., Khan H., Raj B., Schuller B., Steinmetz C., Malloy C., Tzanetakis G., Velarde G., McNally K. (2022). HEAR: Holistic evaluation of audio representations. Proc. Mach. Learn. Res..

[43] Zeinali H., Burget L., Cernocký J. Convolutional neural networks and x-vector embedding for DCASE2018 acoustic scene classification challenge. Proceedings of the Workshop on Detection and Classification of Acoustic Scenes and Events.

[44] Pedregosa F., Varoquaux G., Gramfort A., Michel V., Thirion B., Grisel O., Blondel M., Prettenhofer P., Weiss R., Dubourg V. (2011). Scikit-learn: Machine learning in Python. J. Mach. Learn. Res..

[45] Madsen R.E., Kauchak D., Elkan C. (2005). Modeling word burstiness using the Dirichlet distribution. Proceedings of the International Conference on Machine Learning, Bonn, Germany, 7–11 August 2005.

[46] Řehůřek R., Sojka P. Software Framework for Topic Modelling with Large Corpora. Proceedings of the LREC Workshop on New Challenges for NLP Frameworks.

[47] Bibbó G., Deacon T., Singh A., Plumbley M.D. (2024). The Sounds of Home: A Speech-Removed Residential Audio Dataset for Sound Event Detection. arXiv.

